# Green-Synthesized Silver Nanoparticles Using *Filipendula ulmaria* (L.) Maxim. and *Salvia verticillata* L. Extracts Inhibit Migration and Modulate Redox Homeostasis in Human Breast Cancer Cells via Nrf-2 Signaling Pathway

**DOI:** 10.3390/antiox14040469

**Published:** 2025-04-14

**Authors:** Miloš Matić, Ana Obradović, Milica Paunović, Branka Ognjanović, Vladimir Mihailović, Nikola Srećković, Milan Stanković

**Affiliations:** 1 Department of Biology and Ecology, Faculty of Science, University of Kragujevac, 34000 Kragujevac, Serbia; milos.matic@pmf.kg.ac.rs (M.M.); milica.paunovic@pmf.kg.ac.rs (M.P.); branka.ognjanovic@pmf.kg.ac.rs (B.O.); milan.stankovic@pmf.kg.ac.rs (M.S.); 2 Department of Chemistry, Faculty of Science, University of Kragujevac, 34000 Kragujevac, Serbia; vladimir.mihailovic@pmf.kg.ac.rs (V.M.); nikola.sreckovic@pmf.kg.ac.rs (N.S.)

**Keywords:** silver nanoparticles, breast cancer, redox homeostasis, migration, MMP-9, Nrf-2

## Abstract

Breast cancer is a leading cancer diagnosis for women around the world, with a variable degree of curability. Conventional chemotherapeutic treatments often induce toxicity and damage to healthy tissues, as well as the development of drug resistance, which is why an increasing number of new therapeutic regimens focus on the use of natural products and various modifications of their delivery to target tissues. Silver nanoparticles possess unique physicochemical characteristics, notably their increased surface area, suggesting that they hold significant potential for biomedical applications. This research evaluates the capacity of silver nanoparticles green synthesized with aqueous extracts of *Filipendula ulmaria* (FUAgNPs) and *Salvia verticillata* (SVAgNPs) to alter migration and redox homeostasis in the human breast cancer cell line MDA-MB-231. To determine the values of redox homeostasis parameters, the cells were treated with five different concentrations (5, 10, 20, 50, and 100 μg/mL) for 24 h and 72 h, while to test the migratory potential and concentrations of matrix metalloproteinase-9 (MMP-9) and nuclear factor erythroid 2–related factor 2 (Nrf-2), the cells were treated at two concentrations (5 and 50 µg/mL) for 72 h. The obtained results indicate increased production of superoxide anion radicals, malondialdehyde (MDA), and nitrites after the investigated treatment on MDA-MB-231 cells. The treatments induced only a slight elevation in Nrf-2 levels, which correlates with weak de novo synthesis of reduced glutathione (GSH), suggesting that the tested nanoparticles weaken the inherent antioxidative systems of the tested cells. The migration potential of cells was significantly reduced, and MMP-9 concentration was significantly inhibited. Based on the demonstrated antitumor effect, confirmed by the reduced migratory potential of the examined cells and disrupted redox balance, these nanoparticles have potential for additional investigation with the aim of improving the efficacy of antitumor therapy. Also, FUAgNPs and SVAgNPs possess the capacity to be potentially promising novel chemotherapeutic agents against breast cancer progression and metastasis.

## 1. Introduction

Cancer is one of the most persistent diseases, leading to an increased mortality rate globally. Among various types of cancer, breast cancers rank as the second most prevalent cancer affecting women and the main cause of cancer deaths in women, resulting in over half a million fatalities worldwide each year. Despite the widespread application of chemotherapy, the mortality rate remains high, which emphasizes the need for new therapeutic applications with greater efficacy against cancer cells and high selectivity to avoid damaging healthy tissues [1]. A significant number of breast cancers are hormone-dependent, i.e., hormone-receptor-positive. About 70% of patients have a tumor that expresses estrogen or progesterone, or more precisely, the estrogen receptor or progesterone receptor. In estrogen-sensitive tumors, estrogen is the main factor in tumor growth, which is important for treatment because the main mechanism of action of the used drugs is blocking estrogen. They can act by degrading estrogen receptors or reducing estrogen production in tumor tissue. However, as with chemotherapeutics, resistance is a major problem here [2]. Due to the abundance of epidermal growth factor receptors, increased proliferation occurs, which promotes the growth of transformed cells. Consequently, tumors that have a higher number of these receptors are more resistant to chemotherapy. The goal of treatment is to inhibit these receptors and slow tumor growth [3].

Oxidative stress is one of the main causes of tumor formation, which results from an imbalance between the creation of free radicals and the capacity of cellular antioxidant mechanisms to eliminate these harmful species. Reactive molecules play a crucial role in mediating intracellular signaling pathways and regulating gene expression. Additionally, they play a role in regulating the cell cycle and apoptosis, activating the immune system, and providing defense against pathogens [4]. The action of free radicals and reactive species can lead to epigenetic and genetic changes, which can further lead to genomic instability and initiate the formation and development of tumors [5]. This principle refers to a disruption in homeostasis, indicated by an excess of reactive oxygen species (ROS) and reactive nitrogen species (RNS) on one side, balanced by antioxidant defense mechanisms on the other [6,7]. An increase in the production of these reactive species or a decrease in cellular antioxidant activity results in a disruption in the balance between the generation of free radicals and the effectiveness of antioxidant protection. Such an imbalance can lead to various types of cellular damage, potentially triggering a range of pathological and physiological conditions, including the development of cancer, accelerating the aging process, and ultimately leading to cell death [8].

Matrix metalloproteinases (MMPs) are a class of proteolytic enzymes that are fundamental in the breakdown of extracellular matrix (ECM) components. These enzymes are integral to a variety of physiological functions, including reproduction, tissue repair, and the modulation of inflammatory responses. The activity of MMPs is regulated at multiple levels, including transcriptional control, activation of propeptides, and through the action of tissue inhibitors of metalloproteinases, which serve as natural inhibitors of MMPs. The majority of cancer-related fatalities are attributed to the spread of tumors, a process significantly influenced by metalloproteinases’ activation. MMPs are frequently used as tumor biomarkers [9].

Nuclear factor erythroid 2-related factor 2 is recognized as a principal regulator of the oxidative state within cells, functioning through the interaction of proteins that are ubiquitously expressed. It governs the expression of genes containing the cis-acting antioxidant response element. Chemopreventive agents are known to activate Nrf-2, and the pharmacological activation of this factor has been widely endorsed as a novel approach to cancer prevention. Recent studies indicate that Nrf-2 activity is often heightened in breast cancer cells, and its cytoprotective role could facilitate their proliferation and survival. Nrf-2 is recognized as a key marker in the development and progression of breast cancer. Additionally, it has also been indicated that Nrf-2 is significantly expressed in estrogen receptor-negative breast carcinoma. The dual nature of Nrf-2, acting as both a pro-oncogenic and anti-oncogenic factor in breast cancer cells and healthy cells, suggests the involvement of other factors, including metabolic, proliferative, and angiogenesis genes. Due to its cytoprotective function, Nrf-2 has been recognized as a tumor suppressor [10].

Through continuous research on the effects of various natural and synthetic substances on the growth of different types of tumor cells, it has been observed in our previous research that silver nanoparticles (AgNPs) synthesized using *Filipendula ulmaria* (L.) Maxim. and *Salvia verticillata* L. exhibit significant inhibition of breast cancer cell growth [11]. Therefore, the aim of this research is to examine the potential mechanisms of action of these two AgNP samples on the breast cancer cell line MDA-MB-231 by examining their effects on redox status, migration capacity rate, MMP-9 concentration, and Nrf-2 expression level.

## 2. Materials and Methods

### 2.1. Culturing Cells and Treatment

The human breast cancer cell line, MDA-MB-231, was sourced from the American Tissue Culture Collection (Manassas, VA, USA). These cells were propagated and maintained in complete medium. The cells were cultivated in flasks with medium provided until the cells reached 80% confluence. The cells were seeded into 96-well microplates (10,000 cells per well) and further placed in an incubator with a 5% CO_2_ at 37 °C atmosphere. Following cell adhesion, 100 µL of medium supplemented with different treatment doses (5, 10, 20, 50, and 100 µg/mL) were added in each well, and cells were incubated for 24 h and 72 h. After the end of the treatment period, the superoxide anion radical, nitrite, and glutathione levels were measured. Cells without treatment were used as the control. All experiments were conducted in triplicate for each method.

FUAgNPs and SVAgNPs used in experiments were previously synthesized via a green synthesis approach, using aqueous extracts of *F. ulmaria* and *S. verticillata*, as described in a previous publication [11]. The used silver nanoparticles, FUAgNPs and SVAgNPs, were characterized using UV-Vis, FT-IR, XRPD, SEM/EDS, and DLS techniques and the results were published in the same publication. 

### 2.2. Determination of Superoxide Anion Radical (NBT Test)

The measurement of the superoxide anion radical (O_2_^•−^) concentration in the sample was conducted spectrophotometrically [12], relying on the reduction of nitroblue tetrazolium (NBT) to nitroblue formazan in the presence of O_2_^•−^. The method involved the addition of 20 μL of 5 mg/mL NBT to each well followed by incubating the cells for 1 h at 37 °C in 5% CO_2_. To quantify the formazan production, formazan was solubilized in 20 μL DMSO. The absorbances were measured at 550 nm, and the concentrations of O_2_^•−^ were expressed as nanomoles per milliliter (nmol NBT/mL) in 10^5^ cells.

### 2.3. Determination of NO Concentration (Griess Method)

The measurement of nitrites (NO_2_^−^), a nitric oxide (NO) indicator, was accomplished spectrophotometrically by the Griess method [13]. The concentration of NO_2_^−^ correlates directly with the intensity of the purple color measured at 550 nm. The Griess reaction relates to NO-generated diazonium ion interaction with N-(1-napthyl) ethylenediamine. To prepare the Griess reagent, equal volumes of 0.1% (1 mg/mL) N-1-napthylethylenediamine dihydrochloride and 1% (10 mg/mL) sulfanilamide solution in 5% phosphoric acid were combined immediately prior to application on the plate. After a 10 min incubation, absorbances were measured at 550 nm, and the nitrite concentration was expressed in μmol NO_2_^−^/mL in 10^5^ cells.

### 2.4. Determination of Reduced and Total Glutathione Concentration

The assessment of reduced glutathione is predicated on the reaction between GSH and the sulfide reagent DTNB, resulting in the formation of a yellow compound known as 5′-thio-2-nitrobenzoic acid (TNB) [14]. Following the treatments, 2.5% sulfosalicylic acid was added, and cells were sonicated. A 50 μL amount of the preprepared reaction mixture (1 mM DTNB dissolved in 100 mM phosphate buffer) was added. To measure total glutathione, 50 μL of the reaction mixture (1 mM DTNB, 1 mM NADPH, and 0.7 U glutathione reductase in 100 mM phosphate buffer) was used [15]. The microplates were incubated (10 min in the dark), and the absorbances were measured at 405 nm. The concentration of reduced and total glutathione was expressed in μmol/mL in 10^5^ cells.

### 2.5. Determination of Lipid Peroxidation (TBARS Test)

The assessment of lipid peroxidation products was conducted by measuring the formation of 2-thiobarbituric acid-reactive substances (TBARSs) [16]. Cells were treated with FUAgNPs and SVAgNPs at 2 different concentrations (5 µg/mL and 50 µg/mL). This measurement is based on the reaction between MDA and thiobarbituric acid (TBA) at a temperature of 95 °C. The absorbance of the resulting product, primarily MDA, was measured at 535 nm. The results were expressed as nmol MDA/mg protein.

### 2.6. Determination of Migratory Potential (Transwell Method)

Cell migration was assessed by evaluating the cells’ ability to traverse the pores of polycarbonate membranes (with a pore size of 8 µm; Greiner Bio-One, Switzerland, St Gallen) located at the base of transwell chambers. The migration assay was conducted according to the protocol given by Chen [17].

Cells were treated with FUAgNPs and SVAgNPs at 2 different concentrations (5 µg/mL and 50 µg/mL). Control cells were cultured in medium only. After treatment exposure, the cells were placed in the upper chambers of a new plate (100,000 cells in a final volume of 500 μL). The lower chambers of the control cells contained 750 μL of complete medium. After 6 h of incubation, the remaining cells from the upper surface were completely removed by gentle wiping, while the migrating cells were fixed for 20 min at room temperature in 4% paraformaldehyde and stained with 0.1% crystal violet for 10 min. The membranes were then washed, dried, cut, and placed on 96-well plates. Following the addition of 10% acetic acid, the absorbances were measured at 595 nm. The migration index was determined by taking the absorbance ratio of the treated group and dividing it by the absorbance of the control group, and subsequently multiplying the result by 100 to express it as a percentage.

### 2.7. Measurement of MMP-9 Concentration

MMPs are a class of endopeptidases crucial for cell invasion, as they facilitate the degradation of extracellular matrix components, including type IV collagen and native collagen. Utilizing a kit designed for the quantitative assessment of total MMP-9 concentration allows for the evaluation of cell invasiveness. Prior to the assay, all samples and reagents (Elabscience, ELISA, Houston, TX, USA) were prepared according to the specified instructions. Cells were treated with FUAgNPs and SVAgNPs at 2 different concentrations (5 µg/mL and 50 µg/mL). Both groups of cells (control and treated) after treatment were subjected to trypsinization, followed by centrifugation and three washes with PBS. Subsequently, the cells were sonicated, and the resulting supernatant was utilized in accordance with the protocol described by Obradović et al. [18]. Measurement of absorbance was conducted at 450 nm on a microplate reader. The concentration of MMP-9 was calculated using the standard curve.

### 2.8. Measurement of Nrf-2 Concentration

Samples and reagents from NFE2L2 (Elabscience, ELISA, Houston, TX, USA) were prepared according to the specified instructions. Cells without and with FUAgNP and SVAgNP treatment at 2 different concentrations (5 µg/mL and 50 µg/mL), after 72 h (long-term), were trypsinized, centrifuged with subsequent washing in PBS, and finally sonicated. The resulting supernatant was further used in the protocol described by Obradović et al. [18]. The supernatant in a volume of 100 μL was added to a microtiter plate in which the present NFE2L2 bound to the immobilized antibody. This was followed by incubation for 90 min at room temperature. After that, without a wash, Biotinylated Detection Ab working solution was immediately introduced into each well and incubated for one hour. After rinsing unbound substances, HRP Conjugate working solution was introduced into each well and incubated for 30 min. Substrate solution was introduced into each well, and the samples were incubated for 15 min at room temperature in the dark. After the third incubation, Stop Solution was added to each well in a volume of 50 μL. Absorbance was measured at 450 nm. The absorbance is proportional to the concentration of Nrf-2 and calculated using the standard curve.

### 2.9. Statistical Analyses

All data were analyzed using IBM-SPSS 23 software for Windows (SPSS Inc., Chicago, IL, USA). The results were presented as a mean ± standard error (SEM). The Paired-Sample T test was used to determine statistical significance, with a significance level set at * *p* < 0.05.

## 3. Results

### 3.1. Effects on Redox Status in Tumor Cells

The present study explored the effects of five distinct concentrations (from 5 to 100 µg/mL) of FUAgNPs and SVAgNPs on the MDA-MB-231 human breast cancer cell line. To propose a potential mechanism for their antitumor activity, we investigated the effects of FUAgNPs and SVAgNPs on markers of oxidative stress, particularly the production of O_2_^•−^ and NO_2_^−^, in addition to measuring both reduced and total glutathione levels and the products of lipid peroxidation.

#### 3.1.1. Determination of Superoxide Anion Radical (NBT Assay)

Recent research has highlighted the crucial involvement of ROS and ROS-regulated signaling pathways in tumor invasion. Increased ROS levels have been observed in breast tumors as well as in the adjacent cells of the tumor microenvironment (TME). ROS serve as essential elements within the breast TME, facilitating bidirectional communication among various components and playing complex roles in tumor progression and metastasis [19].

**Figure 1:**
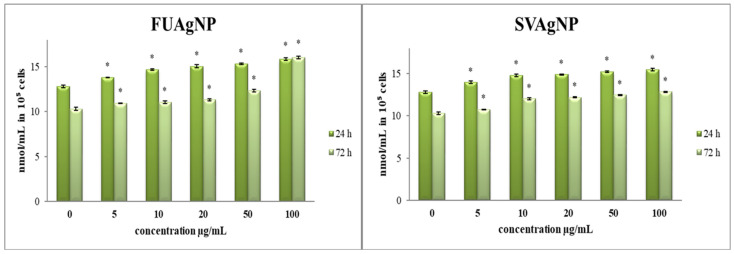
The level of O_2_^•−^ in MDA-MB-231 cells after 24 and 72 h of treatment with FUAgNPs and SVAgNPs. Data represent mean ± standard error: * *p* < 0.05 relative to control.

In comparison to the control, all treatments provoked a notable increase in O_2_^•−^ concentration in tested cells (Figure 1). Given that oxidative stress and the disruption of redox homeostasis are key characteristics of cancer cells, our findings suggest that the observed antioxidant effects may play a crucial role in regulating the progression and viability of cancer cells. The specific antitumor effects are contingent upon the type of tumor and the dosage administered, highlighting the necessity for personalized treatment approaches [20,21].

#### 3.1.2. Determination of Nitrites (Griess Assay)

NO functions as an essential signaling molecule in a range of pathophysiological conditions. Research indicates that NO exhibits both antitumor and protumor effects, depending on the time, level, and tissue [22].

**Figure 2:**
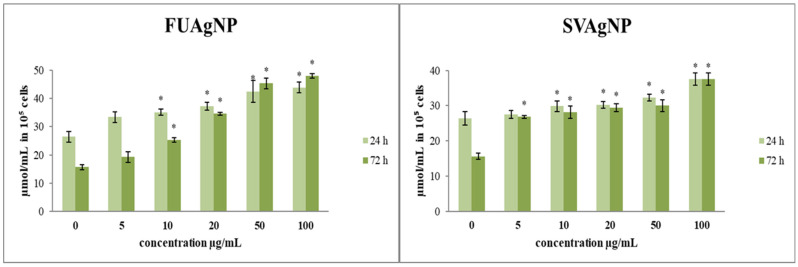
The level of NO_2_^−^ in MDA-MB-231 cells after 24 and 72 h of treatment with FUAgNPs and SVAgNPs. Data represent mean ± standard error: * *p* < 0.05 relative to control.

FUAgNPs and SVAgNPs caused a significant elevation of nitrites in tested cells (Figure 2). Changes in NO production may influence multiple signaling pathways, leading to potential antitumor outcomes. O_2_^•−^ exhibits a strong affinity for NO, a reaction in which highly reactive peroxynitrite (ONOO^−^) is produced, and leads to nitrositive stress [23,24]. Furthermore, most of the concentrations indicated increased production of NO along with notable dose-dependent cytotoxic effects. These results suggest that the examined AgNPs are appropriate for further research aimed at developing new antitumor therapies.

#### 3.1.3. Determination of Total and Reduced Glutathione

**Figure 3:**
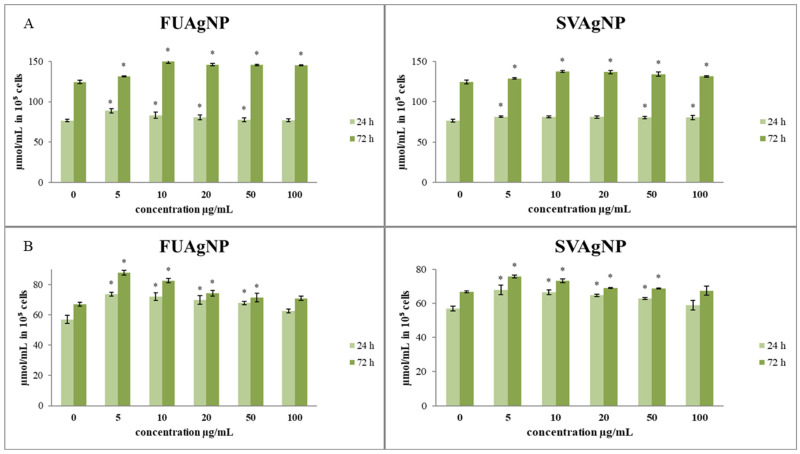
The level of (**A**) total glutathione and (**B**) reduced glutathione in MDA-MB-231 cells after 24 and 72 h of treatment with FUAgNPs and SVAgNPs. Data represent mean ± standard error: * *p* < 0.05 relative to control.

The effects of the treatments on total and reduced glutathione levels in cells are presented in Figure 3. The total glutathione concentrations after short- (24 h) and long-term exposure (72 h) to different concentrations of FUAgNPs and SVAgNPs were increased compared to the control. The increase in total glutathione levels was time-dependent. Both tested AgNPs (FUAgNPs and SVAgNPs) resulted in an increase in GSH levels.

#### 3.1.4. Determination of Lipid Peroxidation

**Figure 4:**
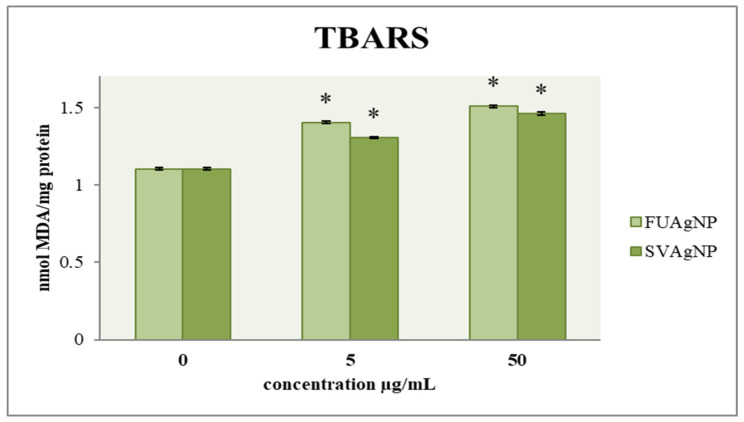
The level of MDA in MDA-MB-231 cells after 72 h of treatment with FUAgNPs and SVAgNPs. Data represent mean ± standard error: * *p* < 0.05 relative to control.

The effect of FUAgNP and SVAgNP treatments on the concentration of malondialdehyde in MDA-MB-231 cells is shown in Figure 4. The results indicate that after 72 h, there is a notable increase in the concentration of MDA in the treated cells compared to the control group, which was dependent on the dose. Numerous studies have suggested that lipid peroxidation could play a role in tumor promotion, as this process leads to the formation of reactive and harmful metabolites. Among the most common and crucial aldehydes resulting from lipid peroxidation is MDA, which can interact with proteins, DNA, and other biomolecules, leading to modifications in their structure and functionality. In recent years, the measurement of MDA levels in tissues and plasma has been extensively utilized in research concerning different cancers, including breast cancer. Several investigations have explored the potential link between lipid peroxidation and the development of breast cancer [25].

### 3.2. Transwell Assay for Cell Migration

**Figure 5:**
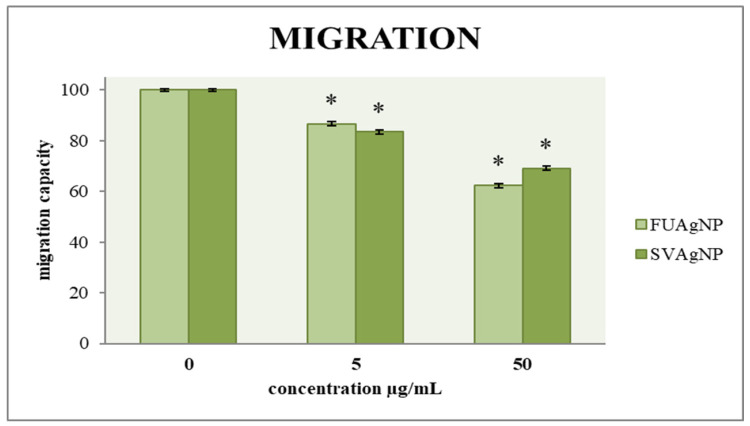
The migration capacity of MDA-MB-231 cells after 72 h of treatment with FUAgNPs and SVAgNPs. Data represent mean ± standard error: * *p* < 0.05 relative to control.

The ability of cells to migrate is a key factor in assessing tumor progression; therefore, an effective antitumor agent must influence cell mobility. A two-dimensional transwell migration assay was conducted to investigate the effects of FUAgNP and SVAgNP treatments on the migration of cells. The data indicates dose-dependent reductions in the migration of MDA-MB-231 cells in response to these nanoparticles compared to the non-treated cells, as shown in Figure 5.

### 3.3. Concentration of MMP-9

**Figure 6:**
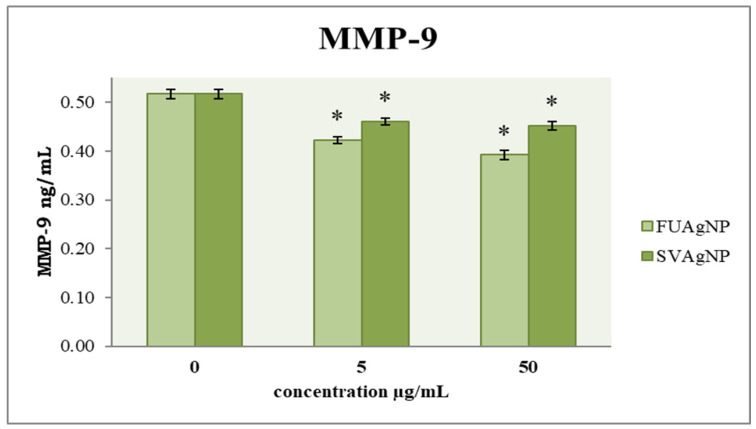
The level of MMP-9 in MDA-MB-231 cells after 72 h of treatment with FUAgNPs and SVAgNPs. Data represent mean ± standard error: * *p* < 0.05 relative to control.

MMP-9 is considered crucial for the metastatic ability of tumor cells. To examine the cells’ migration, an evaluation of their invasive potential was also conducted. The obtained results (Figure 6) indicate that FUAgNPs and SVAgNPs in both concentrations used caused a significant reduction in MMP-9 levels compared to the control. The strongest effect was observed after 72 h of treatment with 50 µg/mL FUAgNPs on MDA-MB-231 cells.

### 3.4. Concentration of Nrf-2

**Figure 7:**
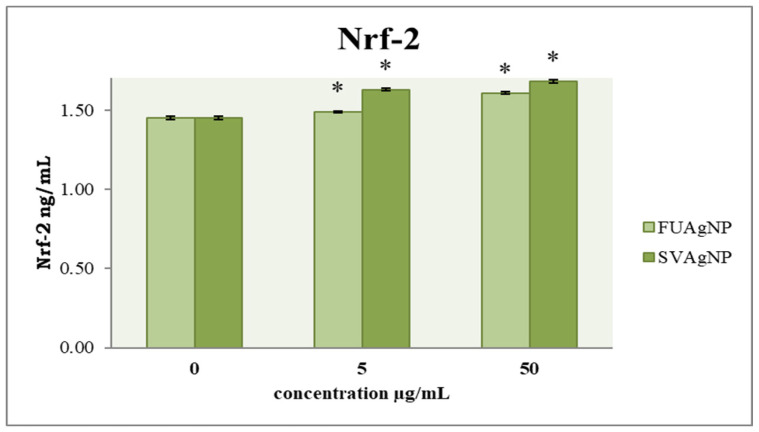
The level of Nrf-2 in MDA-MB-231 cells after 72 h of treatment with FUAgNPs and SVAgNPs. Data represent mean ± standard error: * *p* < 0.05 relative to control.

In healthy tissues, Nfr-2 has been widely recognized as a key mechanism for cancer prevention. Targeting Nrf-2 in the context of cancer progression may offer innovative insights for the development of more effective therapeutic agents. Our results show that FUAgNPs and SVAgNPs in both concentrations used elevated Nrf-2 levels compared to the control (Figure 7).

## 4. Discussion

Cancer is a fatal disease with a complex pathophysiology and one of the most prevalent diseases worldwide in the last few decades. It is a disease influenced by various factors that leads to unregulated proliferation and invasion of altered cells into surrounding tissues. The high cost of drugs and the increased resistance and side effects of current therapeutic approaches have directed research towards examining herbal drugs, which are used in traditional medicine, as an option for finding new chemical combinations for cancer treatment [26]. The primary challenges associated with conventional cancer chemotherapy include a lack of specificity and cytotoxicity, along with the prevalence of multidrug resistance, which frequently leads to treatment failures. Consequently, recent years have seen considerable focus on the advancement of a contemporary discipline known as nano-oncology. This innovative field explores the utilization of nanoparticles for the detection, targeting, and treatment of cancerous conditions [27]. Recently, nanoparticles synthesized using plants have attracted considerable interest due to their special effects, such as biocompatibility, eco-friendliness, and low toxicity. These plant-based nanoparticles have shown promising anticancer activity by targeting cancer cells, disrupting their growth, and inducing apoptosis. Furthermore, they can influence various metabolic pathways within cancer cells, modulating cellular signaling, oxidative stress, and gene expression, which may enhance their therapeutic potential in cancer treatment. Nanoparticles synthesized from *F. ulmaria* and *S. verticillata* are of particular interest due to the bioactive compounds present in these plants [28,29]. These compounds may not only enhance the anticancer potential of the nanoparticles but also improve their compatibility with healthy cells, thus increasing selectivity and reducing side effects. When incorporated into nanoparticles, these bioactive compounds can be more efficiently delivered to cancer cells, enhancing their anticancer effects through targeted interactions with cellular signaling pathways, induction of apoptosis, and modulation of metabolic processes in cancer cells [30]. In our previous study, we showed that FUAgNPs and SVAgNPs induce antiproliferative effects in MDA-MB-231 cells, suggesting significant antitumor capacity [11]. Therefore, in this study, we examined the treatment of FUAgNPs and SVAgNPs on biomarkers of oxidative stress as a possible pathway of their antiproliferative potential. Reactive oxygen and nitrogen species play a vital role in cellular signaling and biosynthetic processes; however, elevated production of these reactive species can result in oxidative stress [31,32]. Oxidative stress is defined by an imbalance between the generation of these harmful radicals and their elimination by antioxidant defenses, potentially causing extensive cellular damage and contributing to the onset of various diseases [33]. Since the superoxide anion radical represents one key ROS element, this study has investigated different concentrations of FUAgNP and SVAgNP treatments on O_2_^•−^ levels in MDA-MB-231 cells after 24 h and 72 h. Our study presented that treatment with the examined AgNPs increased O_2_^•−^ levels in MDA-MB-231 cells compared to the levels in control cells. Both types of AgNPs exhibited pro-oxidative effects during both treatments, and the strongest effects were measured after short-term treatment (24 h).

The reactive oxygen and nitrogen species, along with the oxidative stress they induce, are recognized as mutagenic and carcinogenic due to their capacity to inflict damage on essential macromolecules, including DNA, lipids, and proteins, which can lead to genomic instability and disrupt normal cell growth. The results of the study indicated that AgNPs caused a statistically increased production of ROS compared to the control, which showed that nanoparticles were able to elevate the level of ROS in tumor cells, thus disrupting the redox balance, which could further result in programmed cell death and/or cell growth inhibition previously detected in the treatments [34].

Nitric oxide disrupts the energy supply of cells by damaging mitochondria, and negatively affects cell proliferation, making it a possible therapeutic agent for cancer treatment [35]. However, NO has been indicated to exert an anti- or protumor effect depending on the concentration and exposure time, as well as the type of tumor [36]. Based on our results, we can conclude that the time of exposure and the concentration of treatment are important factors for the mode and intensity of FUAgNPs and SVAgNPs’ effects on nitrite production in human breast cancer cells. That is to say, the effect of the investigated nanoparticles on MDA-MB-231 cells differs when the cells are incubated with treatment for 24 and 72 h. After 24 h, they act to increase nitrite production, while after 72 h, an increase is observed only at higher concentrations, thus demonstrating the dose–time dependence of the treatment depending on the effect. While the majority of research suggests that NO promotes tumor progression, some studies reveal that elevated levels of NO, produced through the transfection of the *iNOS* gene in tumor cell lines, can inhibit growth [37]. These results clearly show that the tested FUAgNPs and SVAgNPs cause an increase in NO production in the tested cells, as one significant antitumor mechanism of the tested silver nanoparticles.

Tumor cells exhibit an increased energy requirement to facilitate their proliferation, resulting in the increased production of ROS in comparison to normal cells. ROS interact with membrane phospholipids, inducing lipid peroxidation. Therefore, their inherent antioxidative capacity is potentiated in order to maintain the integrity of cell structures. Glutathione is a tripeptide molecule composed of three amino acids: cysteine, glycine, and glutamic acid. It plays a pivotal role in the cellular antioxidant defense system and is essential for preventing lipid peroxidation caused by free radicals and oxygen [38].

In breast cancer specifically, GSH concentrations were found to be more than doubled compared to those in normal breast tissue, with even higher levels present in lymph node metastases. Supporting this finding, another investigation noted a marked rise in the levels of oxidized glutathione (GSSG) and total glutathione in breast cancer cells in comparison to the surrounding non-cancerous cells [39]. Our results show a slight elevation in reduced and total GSH levels after the treatments compared to the control, suggesting that the tested nanoparticles debilitate the inherent antioxidative potential of breast cancer cells, making them more sensitive to ROS assaults.

Several studies indicate that heightened cellular levels of glutathione are essential for the initiation and growth of tumors. The increase in GSH levels can be attributed not only to the elevated production of ROS in many tumor cells but also to the activation of GSH synthesis and turnover mechanisms by certain classical tumor promoters. For instance, in tumors with mutated *KEAP1*, there is an increase in Nrf-2 activity and a greater conversion of glutamine to glutamate for GSH production. Additionally, the expression of the cystine/glutamate antiporter, which facilitates the transport of cysteine necessary for GSH synthesis, is significantly upregulated in human tumors [40].

The role of ROS is significant in many cellular processes essential for maintaining cellular homeostasis, but they can also participate in various elements of tumorigenesis, including cell invasion and metastasis [41]. Migration and invasion of tumor cells into the surrounding tissue are essential steps in the progression of tumor metastasis, and many therapeutic approaches are based on reducing the migratory capacity of tumor cells [42]. Metastasis is a complex process where cancer cells evade the main tumor mass and migrate into the surrounding tissues and vascular networks. Therefore, metastatic spread, rather than the primary tumor, is the leading cause of death from malignant diseases. The role of MMPs is crucial in the remodeling processes of healthy tissues, as well as in various pathological conditions. They achieve their role by degrading extracellular matrix stromal proteins and by interacting with cell surface receptors. Cancer progression biomarkers are integral to the processes of cancer diagnosis and prognosis, the monitoring of disease advancement, the prediction of treatment effectiveness, and cancer screening. MMP-9 serves as a crucial molecule in various physiological functions, including reproduction and inflammation, but also assists cancer cells in spreading from the primary tumor. Also, this enzyme plays an important role in cancer cell invasion, and it has been recognized as a potential biomarker for various types of cancer [43].

Various studies have confirmed that AgNPs can inhibit the migration and invasion of tumor cells in a dose-dependent manner. Migration and invasion are significant features of cancer development and progression. Despite the findings that AgNPs can suppress tumor invasion, the underlying mechanism remains unknown. It is theorized that AgNPs can inhibit the protein production of cytokines and growth factors in cancer cells, as well as the enzymatic activity of MMPs [44].

The results presented by Sathishkumar et al. [45] indicate that AgNPs can inhibit the migration of cancer cells and decrease the likelihood of breast cancer spread. Furthermore, data suggest that AgNPs reduced cell migration in addition to inhibiting cancer cell growth by inducing apoptosis. The toxicity, proliferation, and antimetastatic properties of FILE (*Ficus ingens* leaf extract) AgNPs were evaluated using MDA-MB 231 cells, and metastasis of these cells was inhibited in a dose-dependent manner. The FILE-AgNPs generated have exceptional prospects for the treatment of cancer patients and other cancer-related situations [46].

In healthy cells, Nrf-2 is expressed at baseline levels, effectively preventing oxidative stress and regulating the physiological concentrations of ROS. In contrast, cancer cells exhibit overexpression of Nrf-2 linked to several processes: the emergence of drug resistance, angiogenesis, the formation of cancer stem cell phenotypes, and metastasis [9]. Hyperactivation of Nrf-2 is detected in various cell processes including cell survival and proliferation through alterations in cell metabolism and functional adaptations in cancerous states, protecting these cells from apoptosis. Several risk factors are implicated in breast cancer incidence, including sex, aging, estrogen, family history, gene mutations, and unhealthy lifestyle. According to several studies, disrupting the essential antioxidant system at a high level of ROS (but lower than the toxicity threshold) in cells can trigger the incidence and progression of various cancers. However, ROS at a level higher than the harmful threshold can lead to suppression and death of cancer cells. Therefore, precisely identifying molecular mechanisms involving Nrf-2 expression and regulation is helpful in cancer treatment. To deal with the effects of ROS, cells modulate several signaling pathways, which leads to the creation of optimal conditions for their unlimited growth and proliferation. Hyperactivation of the Nrf-2 pathway in cancer cells provides a suitable condition for their growth, proliferation, and drug resistance due to decreasing ROS below the toxicity threshold. Thus, the downregulation of this pathway by various inhibitors to raise the level of ROS can suppress cancer cell development. Besides the cytoprotective role of this pathway, *Nrf-2* can be expressed indefinitely in cancer cells through the gain of mutations. Its hyperactivation accounts for the oncogenic features of cancer cells and triggers cancer cell growth, proliferation, angiogenesis, and chemo/radioresistance [47].

Therefore, targeting Nrf-2, as a crucial molecule for the progression of cancer, offers a novel approach for the development of more effective pharmaceuticals. While redox mechanism-based therapies are recognized for their significant impact on cancer treatment, Nrf-2 plays a crucial role in regulating cytoprotective functions by activating various genes associated with glutathione production and chemoresistance. The tested nanoparticles did not induce a high level of Nrf-2 stimulation, which is expected in the detected states of strong oxidative bursts that the treatments provoked. These data indicate that the tested nanoparticles prevent the activation of Nrf-2-induced antioxidative pathways in MDA-MB-231 cells, which could assist them in adapting to the strong pro-oxidative states. In our study, the concentration of total and reduced glutathione after 24 h and 72 h of incubation with various concentrations of FUAgNPs and SVAgNPs showed only a weak increase in the levels of glutathione compared to the control, suggesting that almost non-altered Nrf-2 levels could be in correlation with low de novo synthesis of glutathione in breast cancer cells in response to oxidative impact. These results could elucidate the potential mechanisms of antitumor activity of the tested nanoparticles in breast cancer cells. The study also reveals that FUAgNPs and SVAgNPs induced a significant increase in MDA production, suggesting strong lipid peroxidation which could be the main deteriorating element in cancer cell integrity disruption.

## 5. Conclusions

In this research, according to previously demonstrated inhibitory effects of the investigated FUAgNPs and SVAgNPs on the proliferation of the various human cancer cell lines, we elucidate some of the possible antitumor mechanisms of these nanocarriers. The obtained data suggest that the tested FUAgNPs and SVAgNPs exert considerable pro-oxidative and antimigratory potential, intensifying the sensitivity of breast cancer cells to reactive oxygen species and oxidative disturbances, while also inhibiting the migration capacity of MDA-MB-231 cells, making them appropriate candidates for different cotreatments with existing chemotherapeutics. The obtained results indicate that the tested silver nanoparticles are convenient candidates for further studies in the development of new chemotherapeutic strategies based on nanocarriers.

## Data Availability

The original contributions presented in this study are included in the article. Further inquiries can be directed to the corresponding author.
